# Drug-carrying microbubbles as a theranostic tool in convection-enhanced delivery for brain tumor therapy

**DOI:** 10.18632/oncotarget.16218

**Published:** 2017-03-15

**Authors:** Pin-Yuan Chen, Chih-Kuang Yeh, Po-Hung Hsu, Chung-Yin Lin, Chiung-Yin Huang, Kuo-Chen Wei, Hao-Li Liu

**Affiliations:** ^1^ Department of Neurosurgery, Chang Gung Memorial Hospital, Linkou Medical Center and School of Medicine, Chang Gung University, Taoyuan 333, Taiwan; ^2^ Department of Biomedical Engineering and Environmental Sciences, National Tsing Hua University, Hsinchu 30013, Taiwan; ^3^ Department of Electrical Engineering, Chang Gung University, Taoyuan 333, Taiwan; ^4^ Medical Imaging Research Center, Institute for Radiological Research, Chang Gung University, Chang Gung Memorial Hospital, Taoyuan 333, Taiwan; ^5^ Department of Neurosurgery, Chang Gung Memorial Hospital, Keelung 204, Taiwan

**Keywords:** convection-enhanced delivery, microbubbles, magnetic resonance imaging, R2 relaxometry

## Abstract

Convection-enhanced delivery (CED) is a promising technique for infusing a therapeutic agent through a catheter with a pressure gradient to create bulk flow for improving drug spread into the brain. So far, gadopentetate dimeglumine (Gd-DTPA) is the most commonly applied surrogate agent for predicting drug distribution through magnetic resonance imaging (MRI). However, Gd-DTPA provides only a short observation duration, and concurrent infusion provides an indirect measure of the exact drug distribution. In this study, we propose using microbubbles as a contrast agent for MRI monitoring, and evaluate their use as a drug-carrying vehicle to directly monitor the infused drug. Results show that microbubbles can provide excellent detectability through MRI relaxometry and accurately represent drug distribution during CED infusion. Compared with the short half-life of Gd-DTPA (1-2 hours), microbubbles allow an extended observation period of up to 12 hours. Moreover, microbubbles provide a sufficiently high drug payload, and glioma mice that underwent a CED infusion of microbubbles carrying doxorubicin presented considerable tumor growth suppression and a significantly improved survival rate. This study recommends microbubbles as a new theranostic tool for CED procedures.

## INTRODUCTION

Convection-enhanced delivery (CED) is a promising technique in interstitial delivery of a drug into brain. It functions by establishing a pressure gradient at the tip of the infusion catheter to create bulk flow to actively “push” the drug into the extracellular space [1, 2]. Compared with the traditional interstitial approach, which relies on drug diffusion, CED is capable of delivering therapeutic macromolecules at a relatively uniform concentration over a larger distribution of the brain [3]. Preclinical studies have been conducted in primates to test the safety of the CED technique [4–7], and the technique has also been clinically tested to enhance the chemotherapeutic agent for treating glioma patients [8, 9] and to enhance neurotrophic factor expression for the treatment of neurodegenerative diseases [10].

The majority of CED studies have indirectly monitored the volumes of distribution by concurrently infusing surrogate tracers in animals: iopanoic acid to enhance computed tomography imaging [3, 9], gadopentetate dimeglumine (Gd-DTPA) to enhance T1-weighted magnetic resonance imaging (MRI) [6, 11–16], or magnetic nanoparticles to enhance T2-weighted MRI [17, 18]. In addition, some studies have used radiolabeled drugs with nuclear imaging monitoring [9]. So far, the concurrent infusion of Gd-DTPA (MW = 938 Da) with the delivered agents or surrogates in CED procedures is the most common method for indirectly monitoring drug or surrogate distribution; it is highly accessible because Gd-DTPA infusion intravenously is now a standard procedure in MRI examinations. However, Gd-DTPA enhances signal for only a short time (the signal typically peaks in intensity at 20–30 minutes and then declines within 1–2 hours through renal clearance [19, 20]). This short signal enhancement period may not be sufficient for the clinical CED procedure since the continuous infusion could persist for 8–24 hours [1, 2].

Microbubbles are lipid-based shells that encap-sulate a stabilized gas (such as hexafluoride, SF6, or perfluoropropane, C3F8). They have a typical size of 2–6 μm, and can circulate in the blood and pass through CNS capillaries. Since microbubbles contain a gas–liquid interface that is highly responsive to ultrasound mechanical stress, microbubbles provide strong ultrasound energy scattering that enables microbubbles to be applied in diagnostic ultrasound as a contrast agent [21–23]. In addition, ultrasound energy interactions with microbubbles have been confirmed to transiently permeate CNS capillaries [24–26].

Microbubbles can potentially be used as a magnetic resonance (MR) contrast agent *in vivo* because of the induction of enhanced local magnetic susceptibility caused by the gas encapsulated in microbubbles. Accordingly, because of the enhanced field susceptibility effect, previous reports have already shown the feasibility of using microbubbles as a contrast medium for CNS imaging in T2- or T2*-weighted MRI [27, 28]. Besides the intrinsic MR sensitivity, modifying the lipid surface of microbubbles enables conjugation via electrostatic and hydrophobic interactions, and such microbubbles have been presented as a drug carrier [29, 30]. We have also developed a technique using these microbubbles to encapsulate and carry chemotherapeutic agents such as BCNU [31, 32] and doxorubicin (Dox) [33, 34]. Since microbubbles may provide MR image contrast and can be designed as a chemotherapeutic drug carrier, we hypothesize that they can potentially be infused with drug-carrying microbubbles during CED and then used to directly monitor the distribution of administered drugs through MRI. A previous study combined the infusion of microbubbles with ultrasound triggering through CED to increase CNS permeation, but did not delineate its feasibility in MRI detectability [35].

In this study, we investigated the feasibility of using microbubbles for monitoring the distribution of an infused drug through MRI, and we propose using drug-carrying microbubbles as a theranostic platform for CED. We employed MR R2 relaxometry to calibrate level changes reflecting the infused microbubble concentration. Dox, a commonly used chemotherapeutic agent, was employed as a test drug. The Dox loading efficiency of microbubbles was evaluated to consider the possibility of using microbubbles as a theranostic platform in CED therapy, and the therapeutic efficiency of CED using an infusion of Dox microbubbles (Dox-MB) was tested on glioma-bearing mice.

## RESULTS

**Figure 1:**
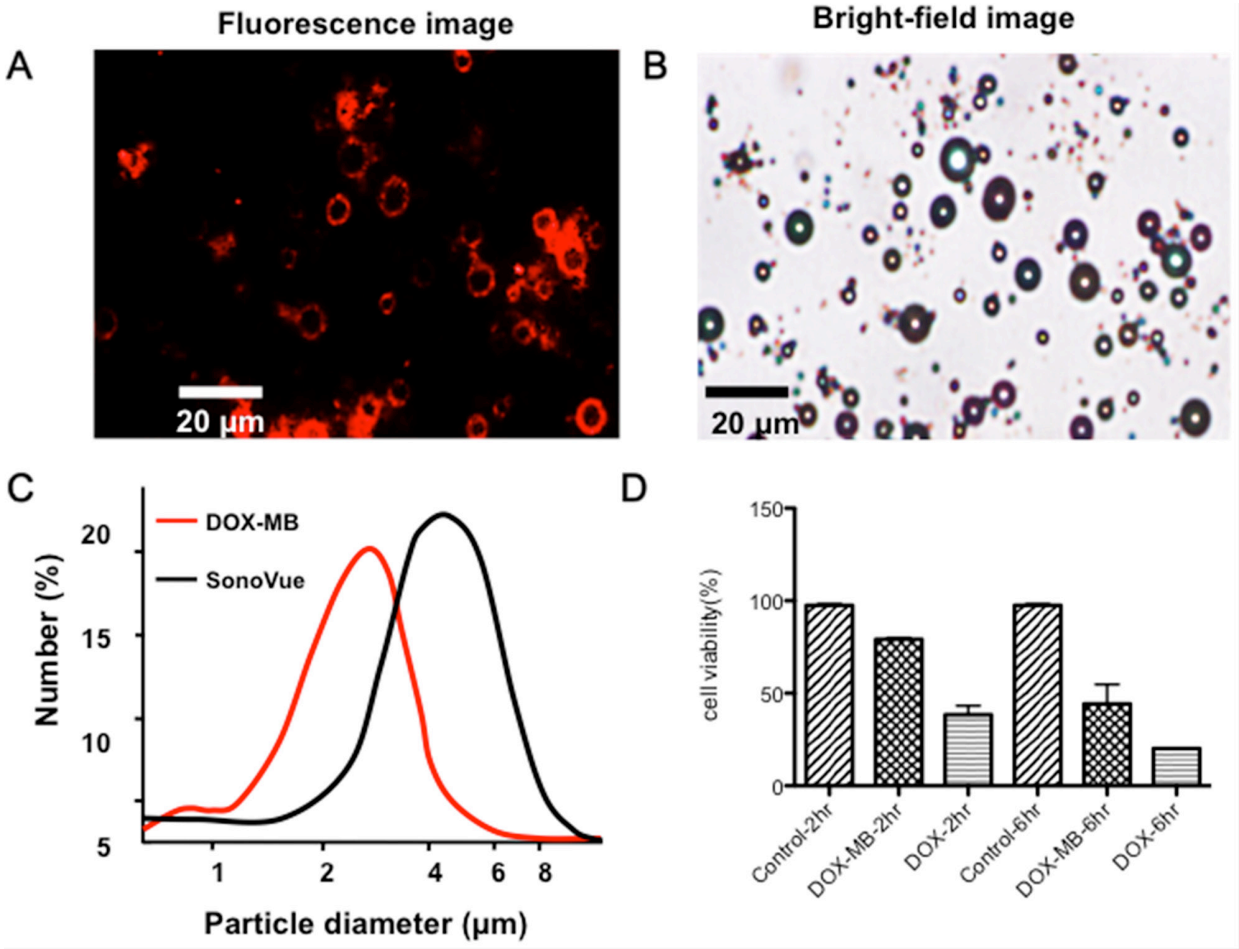
Physical and in-vitro characterization of DOX-loaded microbubbles (DOX-MB) **(A)** Fluorescence image. **(B)** Microscope bright field image of the Dox microbubbles. (Dox-MB) **(C)** Size distribution and structure of Dox-MB and commercially available microbubbles (SonoVue). **(D)** Cell viability test of the Dox-MB versus free Dox.

Figure 1A and 1B shows the fabricated Dox-MB under observation through fluorescence microscopy. The colocalization of the microbubbles in the bright field and fluorescence images indicates strong conjugation of Dox with the bubble surface. The conjugation efficiency of Dox, which was measured by calculating the ratio of bound Dox to the initial Dox amount, was estimated to be 77.6% ± 4.4%. Figure 1C shows the size distribution of Dox-MB compared with commercially available microbubbles (SonoVue). The mean size of the Dox-MB was 2.8 ± 0.9 μm and the mean concentration was (3.4 ± 0.3) × 10^10^ microbubbles/mL. The *in vitro* tested cytotoxicity of the Dox-MB is shown in Figure 1D. Dox-MB presented lower cell toxicity at 2 hours of culturing, with cell viability being 79.22% ± 1.41%, and took 6 hours to reach toxicity similar to that of a 2 hour treatment with free Dox (cell viability = 38.43% ± 8.56% versus 44.27% ± 18.36%). This delayed cytotoxicity of Dox-MB implies that they may release drugs more slowly. This has a potential benefit: more of the drug may be released into tumor cells over the course of *in vivo* delivery.

**Figure 2:**
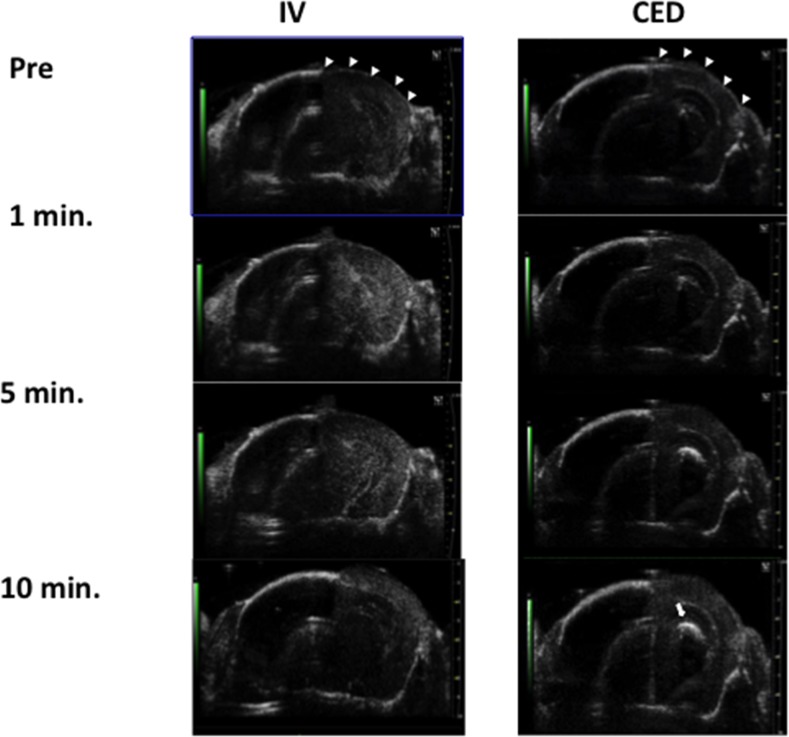
Comparison of microbubble distribution through IV administration (left) and through CED catheter infusion (right) under the observations of small animal diagnostic ultrasound Arrow heads indicate the skull-bone removal area, and the arrow indicates the signal intensity increase due to microbubbles localized at the CED infusion site at the first 10 minutes.

Using in-vivo small-animal ultrasound imaging, we verified microbubble distribution during CED infusion and compared this with the traditional IV administered route. Traditional IV administrations of microbubbles (Figure 2; first column) showed uniformed microbubble distribution for the overall skull-removed brain region (as identified by hyperechoic signals). However, the microbubbles diminished quickly because of liver RES blockage, and the microbubble concentration flowing through the brain tissue rapidly decayed after 5 minutes. By contrast, while the microbubbles were infused through CED (Figure 2, second column), the signals apparently did not decay during infusion but rather presented a hyperechoic increase, which we observed to be highly localized (indicated by arrows). This implies that during CED infusion, microbubbles have a much longer half-life in brain tissue and thus can reveal the location of the infusate at different times.

**Figure 3:**
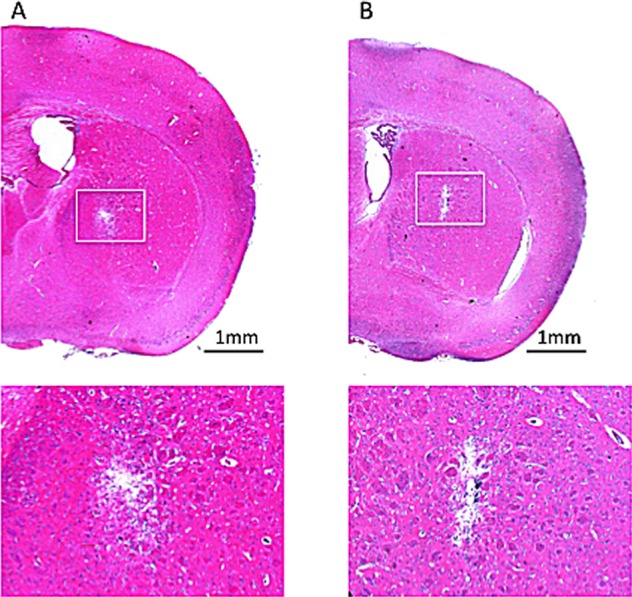
HE staining for histologically identifying potential tissue damage from the CED infused microbubbles **(A)** SonoVue with infused concentration of 0.5 mg/mL. **(B)** In-house fabricated Dox-MB with infusion concentration of 0.5 mg/mL. Bar = 1 mm.

To test the microbubbles’ lipid shells for potential toxicity to CNS tissue, drug-unloaded microbubbles were infused to identify pathological changes. Figure 3 compares the HE stains of normal mouse brain after CED procedure with SovoVue (Figure 3A) for identifying potential histological changes caused by CED microbubble infusions, using SonoVue infusion as a comparison (Figure 3B). It was confirmed that, besides the tract trauma caused by inserting the catheter, no additional tissue damage was caused by the Dox-MB infusion.

**Figure 4:**
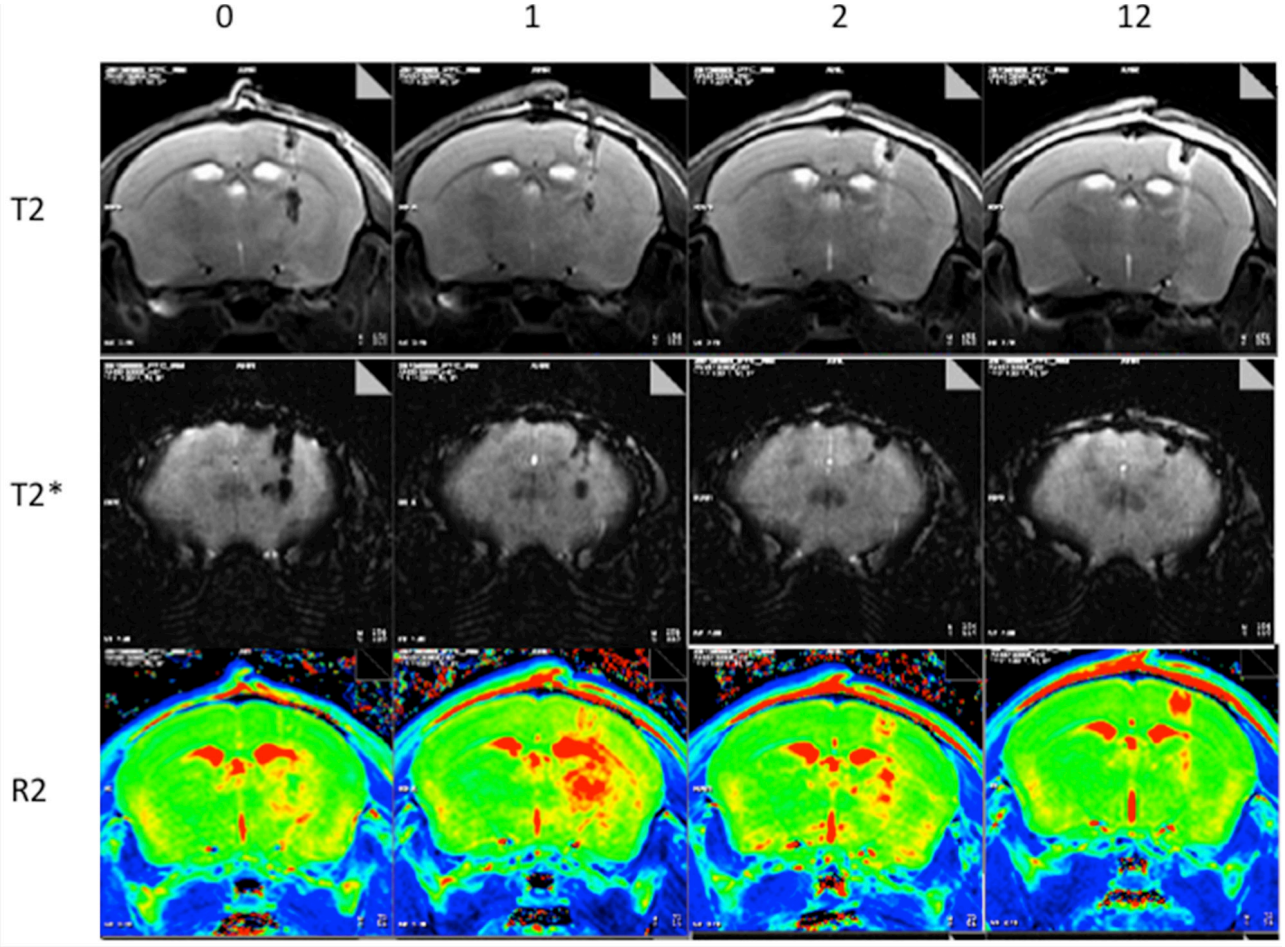
Representative MRI showing longitudinal observations up to 12 hours after Dox-MB infusion (Top: T2 images; Middle: T2* images; Bottom: R2 relaxometry maps).

Figure 4 shows typical MR images of the Dox-MB we assembled, when delivered through CED infusion. The catheter tract can be identified in the T2 weighted images (Figure 4, top row) from the catheter-induced CNS tissue wound (hypointense core) surrounded by edema (hyperintense rim). The T2* images (Figure 4, middle row) show a further increased signal change from the improved sensitivity in magnetic susceptibility caused by the tract wound. However, both the T2 and T2* images showed signal change only along the catheter tract; they were unable to demonstrate the distribution of the infused molecules. By contrast, the R2 map (Figure 4, bottom row) clearly indicates the distribution of CED-infused microbubbles (surround the putamen) and shows that the distribution is much wider than that in the catheter-infused region. Furthermore, the R2 signal plateaued 1 hour after microbubble catheter infusion. The signal can last up to 2 hours after infusion (although with marked R2 signal decay), but we failed to identify an R2 level change 12 hours after CED infusion (apart from accumulation along the catheter tract).

**Figure 5:**
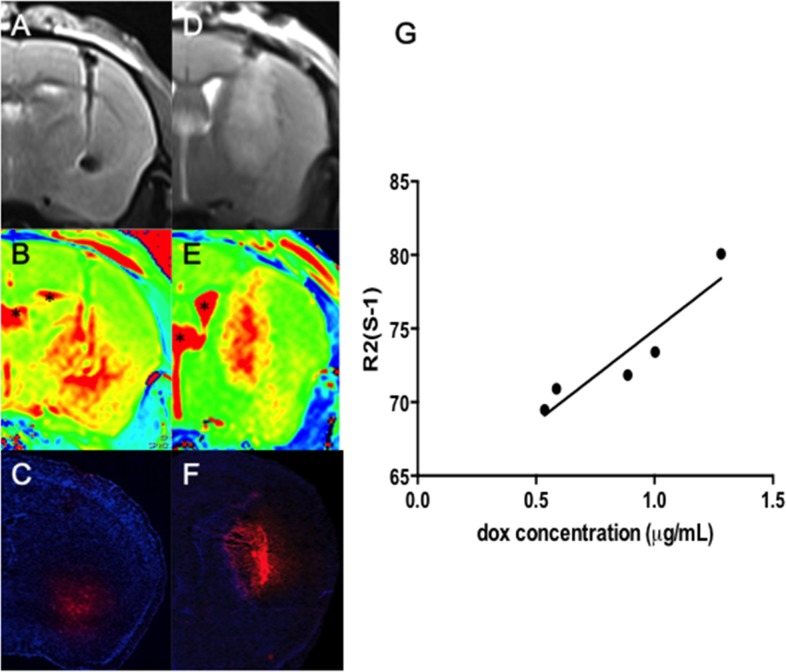
Co-localization of T2 images (A and D) and R2 maps (B and E) with the Dox distribution observed in fluorescence microscopy (C and F) both in normal (A-C) and tumor-implant brain (D-F) (Red: Dox; blue: DAPI for identifying cell nucleus; bar = 1 mm) Regions mark in “*” indicate the ventricle. **(G)** Correlation of the ΔR2 level (in s^−1^) with the CED infused Dox concentration (in μg/mL).

Subsequently, we compared the Dox-MB distri-bution after CED in normal and tumor-implant animals under the observation of MR T2 images (Figure 5A and 5D), R2 maps (Figure 5B and 5E; observed 1 hour after the microbubble infusion) and with the distribution compared with the Dox-emitted fluorescence microscopy (Figure 5C, 5F). The fluorescence distribution emitted from Dox both highly correlated with MRI R2 maps in normal (Figure 5B) and tumor-implant brain (Figure 5E), while excluding the R2 signal-saturating area in ventricle due to distinct R2 relaxometry characteristics of CSF to brain tissue (denoted by “*” in figures). The correlation between the R2 map change and the measured Dox concentration was also seen by measuring the peak R2 level in each infused animal, with the quantitated Dox deposited near the infused tip end of the brain tissue, as well as the contralateral site as a reference (see Figure 4G; n = 5).

In five animal measurements, the Dox concentration ranged from 0.5 to 1.3 μg/mL at the infusion site. This corresponds to an R2 value change of 21–35 s^−1^ at the infusion hemisphere. The estimated correlation between an R2 change and measured Dox concentration was high (r = 0.86), implying that the drug concentration when using microbubbles as a carrier could be directly estimated from R2 maps. These observation supports that MRI R2 map can reveal not only the distribution of Dox-MB, but also can quantitatively estimate the concentration of infused Dox in the brain.

**Figure 6:**
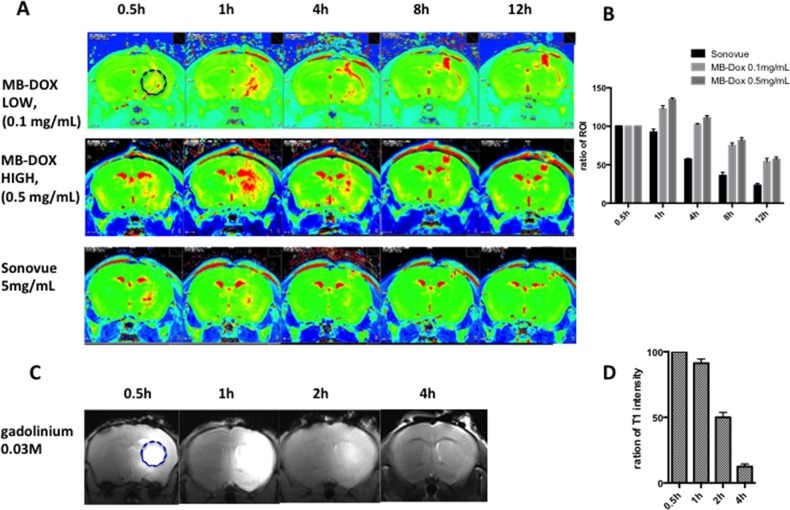
(A) Representative images of the longitudinal R2 map observation of the commercial microbubbles (bottom row) and the in-house fabricated Dox microbubbles under various infusion doses (top row: 0.1 mg/mL; middle row: 0.5 mg/mL) **(B)** Comparison of longitudinal signal intensity changes of central area ROI in R2 map at different time points for CED of the two types of microbubbles (n = 3 in each group). **(C)** Representative T1 image showing the longitudinal fading of CED of the gadolinium contrast medium (Gd-DTPA). **(D)** Ratio changes of central ROI for CED of Gd-DTPA (n = 3).

Figure 6A shows longitudinal R2 level changes of the CED-infused 5 μL Dox microbubbles (with infusions at concentrations of 0.1 mg/mL and 0.5 mg/mL). To compare the R2 detectability of the Dox and SonoVue microbubbles, we also infused SonoVue through CED and monitored the results. Infusing Dox microbubbles at various doses showed a clear R2 level change, where greater R2 intensity changes corresponded with higher concentrations of Dox microbubbles. The R2 map presents a longitudinal change, which implies that longitudinal monitoring of the Dox distribution through MR relaxometry is feasible. We noted that the peak R2 level change under various concentrations of Dox microbubble infusions consistently occurred approximately 1 hour after the CED infusion, with the R2 signal change lasting up to 12 hours. At 8–12 hours after infusion, an R2 level increase was observed only near the tip, close to the cortex; this implies that the remaining microbubbles (or their debris) were refluxed along the infusion tract and accumulated at the tract entry because of the buoyancy effect. The distribution of R2 change could be also identified for SonoVue (at a concentration of 5 mg/mL), but the SonoVue infusion had a relatively short observable R2 duration (the R2 signal vanished after 4 hours), and the R2 signal increased only near the tip end of the infused tract. Furthermore, the distribution of SonoVue in the brain was considerably limited, compared with the distribution of Dox microbubbles.

The comparison of the R2 observation lifetime is clearer in Figure 6B, which depicts an analysis of the R2 level change at the selected ROI (see Figure 6A; a 2-mm circular ROI was selected with the cannula tip end as the center). The estimated half-life, defined by the time to conduct a 50% R2 signal decay, was 4 hours for SonoVue and 12 hours for the Dox microbubble infusion, showing a significantly prolonged half-life for MRI monitoring of the CED-infused bubbles.

For comparison, 5 μL of a clinical contrast agent Gd-DTPA with a concentration of 0.03 M was infused through CED and a serial MR T1 image was recorded (Figure 6C). The intensity of the ROI surrounding the center of infusion was compared at different times. The half-life for this agent was approximately 2 hours.

**Figure 7:**
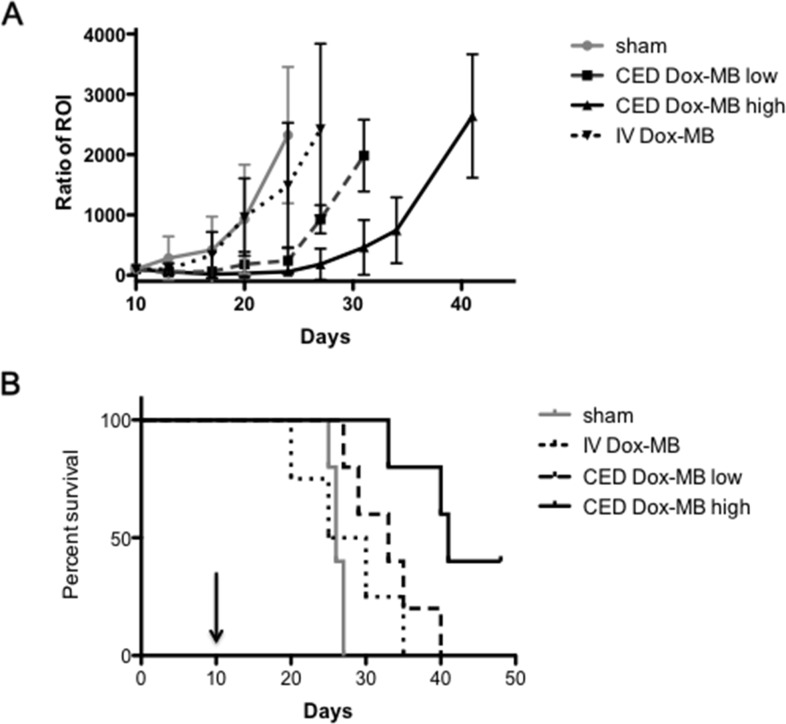
Tumor progression and survival analysis **(A)** Comparison of tumor progression, represented by luminescence intensity detected by the IVIS after the sham procedure (n=4), IV administration of 50uL of Dox-MB (IV Dox-MB; n = 5) and Dox-MB infusion through CED at concentrations 0.1 mg/mL (CED Dox-MB, low; n = 5) and 0.5 mg/mL (CED Dox-MB, high; n = 5). **(B)** Corresponding Kaplan–Meier plot showing animal survival improvement from CED of Dox-MB. Arrow indicates the date to implant tumor cells into animals.

The therapeutic efficacy of the Dox microbubbles infused through CED was then evaluated. With the 5uL of 0.1 mg/mL and 0.5 mg/mL Dox-MB infusions, 19 mice bearing U87 glioma cell implantations were tested (see Figure 7). Figure 7A shows the tumor progression through *in vivo* imaging system (IVIS) observation among various study groups. Animals treated with a sham 5uL microbubbles infusion and with intravenous administration of 50 μL of 0.5 mg/mL Dox-MB both showed a 500-fold increase in tumor progression according to the intensity of luminescence from Day 10 to 17. However, for the treatment groups, this took 26 days for the low dose group and 31 days for the high dose group. The corresponding Kaplan–Meier survival analysis is shown in Figure 7B. The mean survival time for untreated animals and Dox-MB IV-administration group was observed to be 26 and 27.5 days respectively. Infusing Dox microbubbles at a concentration of 0.1 mg/mL extended mean survival to 33 days, whereas increasing the concentration to 0.5 mg/mL extended mean survival to 41 days. The procedure of infused Dox microbubbles through CED not only extended our capabilities for longitudinally monitoring therapeutic agents in the brain through MRI but also provided effective brain tumor treatment.

**Figure 8:**
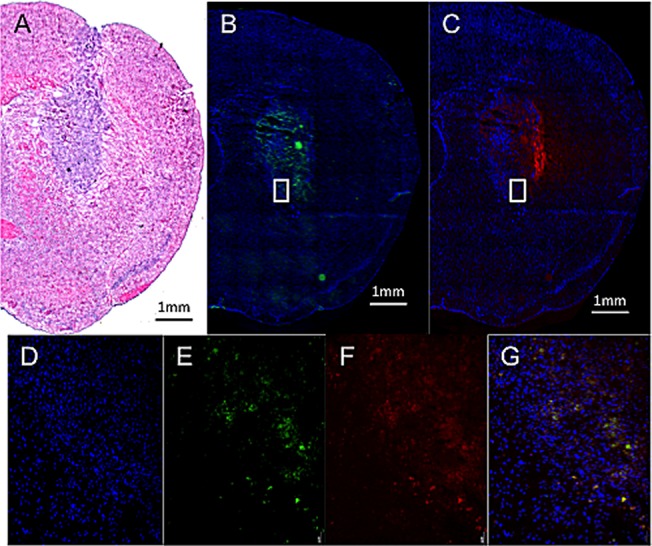
Fluorescent microscopic observation of Dox-MB in tumor-implant brain after CED infusion **(A)** H&E stain showed sizable tumor with hyper-cellularity in putamen. **(B, C)** Fluorescent microscopy showed the distribution of fluorescent-tagged MB shell (in green), with the distribution similar to the Dox (in red). **(D-G)** Zoomed picture of the cell nucleus (in blue), fluorescence emitted from MBs (in green), Dox (in red), and co-localization of the three. Bar = 1 mm.

Figure 8 provides evidence to reveal that MBs (tagged with fluorescent dye; in green; Figure 8B) indeed carried the Dox (in red; Figure 8C) and penetrated into deep intracellular space away from the catheter and was endocytosed by glioma cells (Figure 8D–8G). This supports that MBs can be well delivered through the whole tumor area, and can also support the proposed scheme that combining CED infusion with drug-carrying MBs can deliver drugs into tumors.

## DISCUSSION

### Significance of this study

This study presents, for the first time, the use of microbubbles as an imaging-contrast monitoring agent as well as a drug-carrying vehicle in CED. We show that microbubbles provide excellent detectability through MRI relaxometry and can represent drug distribution during CED infusion. Compared with the traditional gadolinium contrast medium with a half-life of 1–2 hours (Figure 6D) [19, 20], microbubbles provide an extended observation period of up to 12 hours (Figure 6B). Furthermore, where previously the gadolinium contrast medium could be used only as a surrogate agent for indirect monitoring of drug distribution during CED, drug-carrying microbubbles can facilitate direct monitoring, not only of the drug distribution but also of semi-quantitative measurements of the drug in the brain. For lesions such as brain tumors that have contrast-enhancement characteristics, the distribution of the gadolinium contrast medium in the infusate may be disturbed by the lesions and not represent drug distribution. Thus, this study developed microbubbles as a theranostic tool in CED procedures.

### Drug-carrying capability

Microbubbles have long been employed as a thera-nostic agent in ultrasound-imaging-guided drug delivery. The common approach is to encapsulate therapeutic molecules inside a 100 nm liposome structure and perform the conjugation with 2–5 μm microbubbles [36–40]. However, the decoupling of liposome–micro-bubble bonds is easily catalyzed and occurs quickly; consequently, the direct MRI monitoring of the microbubbles may not fully represent the location of the drug, particularly for a long infusion time in the CED procedure (8–24 hours in clinical tests).

We employed a novel bubble fabrication procedure, using not liposomes but Dox mixed directly with a lipid through our previously developed synthesis approach [32, 33]. Dox was linked to the lipid structure (this can be confirmed from Figure 1A, where the emitted red fluorescence is colocalized with the lipid from Figure 1B). An advantage of the formula is that Dox can be preserved in the microbubbles during diffusion, preventing CNS cell damage that the free drug would normally cause. Dox -MB may also enter further into tumor cells because the endocytosis of tumor cells is more effective than that of normal CNS cells.

### Characteristics of our microbubbles

In this study, we compared SonoVue microbubbles with the microbubbles we synthesized considering their differences in MRI R2 relaxometry and observation duration. Our microbubbles allow a longer observation period (up to 12 hours) than the SonoVue microbubbles do (4 hours). This may result from the microbubbles’ size: the mean size of our bubbles was approximately 2 μm compared with SonoVue's 5 μm. Larger microbubbles present greater concerns for bubble stability, because larger lipid shells tend to collapse through catalysis and unbalanced surface tension, leading to rapid macroscopic folding events, bubble disruption, or bubble resolution [41]. By contrast, our microbubbles have greater structural stability from their smaller size, and a bubble accumulation plateau can be observed after infusion. This might explain the difference between the dynamics of SonoVue (with its monotonic signal decreasing longitudinally; see Figure 6B, black bar) and those of our microbubbles (which increased to a concentration plateau and then descended; see Figure 6B, gray bars).

### Therapeutic level of Dox

Previous Dox anticancer studies have provided a comprehensive understanding of the Dox concentration required to be therapeutic. Clinically, it has been reported that intratumoral Dox concentrations reaching 819 ± 482 ng/mL tissue correlate with partial or complete responses in breast cancer patients [42]. Our study shows that the proposed Dox-MB provide an excellent drug-loading efficiency of 0.78 ± 32 mg/mL. We observed that the glioma-implanted mice responded well to the infused Dox-MB, and the measured local concentration of Dox infused through CED reached 0.6–1.3 μg/mL locally. This implies that the microbubbles can carry a sufficiently high concentration of Dox into the brain through CED, and the delivered amount of Dox can reach therapeutic levels. This is supported by the therapeutic efficacy presented in Figure 7: the 0.5 mg/mL Dox-MB infusion retarded tumor progression and prolonged animal survival.

In conclusion, CED is an efficient system for delivering chemotherapeutics to brain tumors. Lipid-based microbubbles provide imaging information for both the distribution and quantity of the infusate, and may circumvent confusion with enhanced lesions. Moreover, loading the microbubbles with drugs has demonstrable theranostic value for brain tumor treatment.

## MATERIALS AND METHODS

### Microbubbles

The lipid shell of the Dox-MB was composed of 1,2-distearoyl-sn-glycero-3-phosphocholine (Avanti Polar Lipids, AL, USA), 1,2-distearoyl-sn-glycero-3-phospho-rac-glycerol sodium salt (Avanti Polar Lipids), and 1,2-distearoyl-sn-glycero-3-phosphoethanolamine-N-[methoxy(poly (ethyleneglycol))-2000] (Avanti Polar Lipids) at a molar ratio of 21:21:1, homogeneously dissolved with chloroform. The chloroform was then removed using an evaporator (R-210, Büchi Labortechnik AG, Flawil, Switzerland), and glycerol PBS (5 wt%) and Dox (1 mg) were mixed with the dried lipid film. The solution was degassed. Subsequently, the samples were refilled with perfluoropropane (C3F8). After intensive shaking in an agitator for 45 seconds, Dox-MB were formed. They were placed on ice for 30 minutes to stabilize the microbubble structures before use. For some batches of the microbubble synthesis, fluorescein amidite (FAM) tagged lipid materials (Sigma Aldrich, MO, USA) were employed for the observation under fluorescent microscopy.

We estimated the payload of Dox on the synthesized bubbles. The supernatant from a Dox-MB suspension was collected as an unloaded Dox-MB complex (free Dox). The Dox-MB were re-suspended with PBS, subjected to sonication for 5 minutes to completely destroy the microbubbles, and centrifuged at 11,000 *g* for 2 minutes. The final precipitate was re-suspended with DDW as a loaded Dox-MB complex. Subsequently, both the free Dox and loaded Dox complex were subjected to a nitrification reaction followed by ICP-AES estimation. The loading efficiency of Dox on the microbubbles was calculated as

$$\text{Dox loading efficiency}(\%)=\frac{W_{\text{load}}}{W_{\text{load}}+W_{\text{free}}}\times 100\%$$ (1)

where W_load_ is the amount of Dox loaded on the microbubbles, and W_free_ is the amount of Dox that was not encapsulated in the microbubbles.

For reference and comparison with our microbubbles, we employed the commercially available SonoVue SF_6_-filled ultrasound microbubbles (2–5 μm, 10 μL/mouse; Bracco, Milan, Italy).

### *In vitro* cytotoxicity test

A cell culture with a concentration of 10^4^ U87MG-Lu cells was placed in each well of a 96-well plate and incubated at 37°C with 5% CO_2_ for 24 hours. Subsequently, the U87MG-Lu cells were cocultured with normal saline, free Dox, or Dox microbubbles (concentration calibrated to 100 μM) for 2 hours or 6 hours to compare their acute cytotoxicity responses. After coculturing, the medium (containing sample) of the cells was moved and refilled with fresh culture medium. The cell viability and proliferation were measured with the alamarBlue reagent (AbDSerotec, Oxford, UK).

### Animal procedures

All animal experiments conducted in this study were approved by the Institutional Animal Care and Use Committee (IACUC), Chang Gung University, and conducted in accordance with its experimental animal care guidelines. Pathogen-free male NU/NU mice (5–7 weeks old, 20–25 g) from BioLASCO (Taiwan) were housed in a controlled environment with all experiments approved by the IACUC. A total of 15 normal mice and 26 glioma-cell-implanted mice were employed in this study.

In the glioma mice, U87MG-Lu glioma cells were cultured at 37°C with 5% CO_2_ in MEM with 10% fetal bovine serum and 1% penicillin/streptomycin (Invitrogen). To implant the U87MG-Lu cells, the animals were anesthetized with 2% isoflurane gas and immobilized on a stereotactic frame. A sagittal incision was made in the skin overlying the calvarium, and a 27G needle was used to create a hole in the exposed cranium 1.5 mm anterior and 2 mm lateral to the bregma. Five microliters of U87MG-Lu cell suspension (1×10^5^ cell/μL) was injected at a depth of 3 mm from the brain surface over a 5-minute period, and the needle was withdrawn over 2 minutes. Brain growth was monitored through MRI for 10 days after implantation.

### CED procedure

The CED procedure was similar to that described in [43]. In brief, infusion cannulae were fabricated with silica tubing (Polymicro Technologies, Phoenix, AZ) fused to a 0.1 mL syringe (Plastic One, Roanoke, VA) with a 0.5 mm stepped-tip needle that protruded from the silica guide base. The syringes were loaded with a liposomal drug (0.04 mg/μL) and attached to a microinfusion pump (Bioanalytical Systems, Lafayette, IN). The syringe with a silica cannula was mounted onto a stereotactic holder and then lowered through a puncture hole made in the skull (for tumor-implanted animals, the infusion occurred at the same region in the caudate putamen where tumor cells had previously been injected). The microbubble solution was infused at a rate of 1 μL/min until a volume of 5 μL had been delivered, and the cannulae were removed 2 minutes afterwards, for a total infusion time of 7 minutes.

### MRI procedure

MRI images were acquired on a 7-T magnetic resonance scanner (Bruker ClinScan, Germany) with a four-channel surface coil used on the top of the mouse brain. After the CED procedure, the anesthetized animals were placed in an acrylic holder and positioned at the magnet center. T2*-weighted imaging sequences were acquired to highlight the magnetic susceptibility effect caused by the infused microbubbles (pulse repetition time (TR)/ echo time (TE) = 30 ms/18 ms; flip angle = 40°; slice thickness = 0.6 mm; matrix size = 256 × 384; FOV = 80 × 130 mm^2^).

R2 relaxometry was performed by obtaining a multiple-TE spin-echo sequence three times with the following parameters: TR = 3860 ms, TE = 8/14/28/57/85/228 ms, matrix size = 128 × 256, FOV = 38 × 76 mm^2^, and slice thickness = 1.4 mm). The obtained multiple-TE images were processed using MATLAB to calculate the R2 value (1/T2) by fitting an exponential curve of the signal intensities as a function of echo time for each pixel [44]. ROIs were set at the tumor site in coronary slices to calculate the average tumor R2 value. Subsequently, color-coded R2 maps were generated. For comparison with the traditional contrast-enhanced T1-weighted imaging detection, Gd-DTPA (Magnevist, Berlex Laboratories, Wayne, NJ) was administered through CED. T1W1 images were acquired using a gradient echo FLASH sequence with the following imaging parameters: TR/TE = 230 ms; FOV = 30 × 17.82 mm^2^; in-plane resolution = 256 × 256 pixels; slice thickness = 0.8 mm; flip angle = 70°.

### Small animal ultrasound imaging

A small animal ultrasound imaging system (Vevo 2100, VisualSonics, Toronto, Canada) was employed to confirm the microbubble distribution from the CED infusion. The hair on the skin over the tumor was clipped, 2 × 2 mm^2^ of the cranial bone of the observed hemisphere was removed, and acoustic gel was applied to provide ultrasound energy coupling. The array transducer had a central frequency of 18 MHz, with axial and lateral resolutions of 75 μm and 165 μm, respectively. The focal length was 8 mm with MI = 0.2. Real-time imaging was performed at a frame rate of 10 Hz (corresponding to a temporal resolution of 100 ms).

### Quantification of Dox release into CNS tissues

After undergoing CED with an infusion of Dox microbubbles, the animals were sacrificed and brain tissues around the infusion center were collected and weighed. The Dox was extracted with the addition of 2 mL of HCl (2 M, at 4°C), and the extracted solution was centrifuged at 15,000 *g* for 15 minutes in a SIGMA 3-30K (Heraeus Co., Germany). The supernatant of the sample was then collected, filtered through a 0.22 μm filter, diluted with a mobile phase solution, and analyzed using high performance liquid chromatography (HPLC) with a UV detector (S1125, Sykam GmbH, Germany). The mobile phase solution consisted of 50 vol% DDW diluted with HPLC-grade methanol in DDW. A column packed with RP-18 (Alltima C-18 3u, Alltech, IL, USA) was used with a detection wavelength of 256 nm and a flow rate of 1.0 mL/min. The amount of Dox was analyzed to determine the area under its peak at a retention time of 3.6 ± 0.2 minutes. The Dox concentration was expressed per gram of tissue.

### Tumor progression monitoring

In an efficacy study, a Spectrum IVIS (Caliper, Hopkington, MA) was used to observe tumor growth. D-luciferin (3 mg/mouse) was intraperitoneally injected into mice before imaging, and a luminescent signal from the tumor was obtained by the IVIS 8 minutes later. Images were taken twice a week for 7 weeks.

### Histological examination

Histopathology was performed on 10 μm sections of paraformaldehyde-fixed, paraffin-embedded brains. Slides were placed in hydrochloric acid–potassium ferrocyanide solution for 30 minutes at room temperature. The slides were counterstained with nuclear fast red for 5 minutes. Brain tissue damage and tumor progression were evaluated using hematoxylin and eosin staining. The distribution of Dox was assessed through red fluorescence microscopy imaging.

### Statistical analysis

Statistical significance was calculated using either a two-tailed unpaired *t* test The Kaplan–Meier method was used for survival analysis. Statistical significance was assumed at p < 0.05.
